# A Sec-Dependent Secretory Protein of the Huanglongbing-Associated Pathogen Suppresses Hypersensitive Cell Death in *Nicotiana benthamiana*

**DOI:** 10.3389/fmicb.2020.594669

**Published:** 2020-11-30

**Authors:** Chao Zhang, Peixiu Du, Hailin Yan, Zongcai Zhu, Xuefeng Wang, Weimin Li

**Affiliations:** ^1^Biotechnology Research Institute, Chinese Academy of Agricultural Sciences, Beijing, China; ^2^College of Plant Science, Tarim University, Alar, China; ^3^Citrus Research Institute, Southwest University, Chongqing, China

**Keywords:** *Candidatus* Liberibacter asiaticus, sec-dependent secretory protein, hypersensitive cell death, small heat shock protein, virulence factor

## Abstract

“*Candidatus* Liberibacter asiaticus” (CLas) is a phloem-restricted Gram-negative bacterium that is the causal agent of citrus huanglongbing (HLB). In this study, we identified a CLas-encoded Sec-dependent secretory protein CLIBASIA_04405 that could contribute to the pathogenicity of this bacterium. The gene expression level of CLIBASIA_04405 was significantly higher in citrus than in psyllids. Transient overexpression of the mature CLIBASIA_04405 protein (m4405) in *Nicotiana benthamiana* leaves could suppress hypersensitive response (HR)-based cell death and H_2_O_2_ accumulation triggered by the mouse BAX and the *Phytophthora infestans* INF1. An alanine-substitution mutagenesis assay revealed the essential of amino acid clusters EKR^45–47^ and DE^64–65^ in cell death suppression. Challenge inoculation of the transgenic *N. benthamiana*-expressing m4405 with *Pseudomonas syringae* DC3000Δ*hopQ1-1* demonstrated the greatly reduced bacterial proliferation. Remarkably, transcriptome profiling and RT-qPCR analysis disclosed that the gene expression of six small heat shock proteins (sHSPs), a set of plant defense regulators, were significantly elevated in the transgenic m4405 lines compared with those in wild-type *N. benthamiana*. In addition, the transgenic m4405 lines displayed phenotypes of dwarfism and leaf deformation. Altogether, these data indicated that m4405 was a virulence factor of CLas.

## Introduction

Citrus huanglongbing (HLB), also known as citrus greening, is the most devastating worldwide disease of citrus at the moment and greatly damages the citrus industry by shortening lifespan of trees and reducing fruit yield and quality (Bové, 2006; Gottwald, 2010). The disease is associated with “*Candidatus* Liberibacter asiaticus” species that are phloem-limited, fastidious alpha-proteobacteria (Bové and Ayres, 2007; Wang and Trivedi, 2013). To date, three *Ca.* Liberibacter asiaticus species have been identified to be the causal agent of HLB, including “*Candidatus* Liberibacter asiaticus” (CLas), “*Ca.* L. africanus” (CLaf), and “*Ca.* L. americanus” (CLam) (Jagoueix et al., 1996; Wang et al., 2017). Among them, CLas is transmitted among citrus by *Diaphorina citri* Kuwayama [Asian citrus psyllid (ACP)] and is the most widespread and virulent species (Gottwald, 2010). So far, no citrus variety has been identified that possesses immunity to CLas (Folimonova et al., 2009; da Graça et al., 2016; Martinelli and Dandekar, 2017; Zheng et al., 2018). Given its serious impact on citrus production, CLas attracts increasing attention. However, the pathogenicity of CLas remains largely unknown mostly owing to its fastidiousness.

Bacterial pathogens often secrete proteins (also called effectors) that contribute to their pathogenicity (Hayes et al., 2010; Dou and Zhou, 2012). It is known that many Gram-negative bacteria depend on a type III secretion system (T3SS), T4SS, or T6SS to deliver effectors into the host cells and thus allow the manipulation of host cellular pathways to benefit the pathogen (Yamane et al., 2004; Wang et al., 2011). As an intracellular bacterium, CLas does not possess this conserved secretion mechanism (Duan et al., 2009). However, like phytoplasmas, another group of phloem-dwelling intracellular bacteria (MacLean et al., 2011; Sugio et al., 2011), CLas was inferred to secrete effector proteins to host cells via a Sec-dependent secretion system (Duan et al., 2009; Prasad et al., 2016).

A significant number of CLas-encoded proteins have been experimentally validated to be Sec-dependent secretory proteins (Prasad et al., 2016; Zhang et al., 2019), and their potential roles in CLas pathogenesis are beginning to be revealed. For example, CLIBASIA_03875 and CLIBASIA_04025 suppress programmed cell death (PCD) (Zhang et al., 2019; Pang et al., 2020), CLIBASIA_05315 targets the chloroplasts and induces cell death in *Nicotiana benthamiana* (Pitino et al., 2016, 2018) as well as physically interacting with the papain-like cysteine proteases (PL) and inhibiting their protease activity in citrus (Clark et al., 2018). In addition, a subset of Sec-dependent secretory proteins were deduced to be candidate effectors that play roles during CLas infection using a *Tobacco mosaic virus*-based expression system or a strategy for the analysis of temporal and spatial gene expression (Shi et al., 2019; Ying et al., 2019; Li et al., 2020). Recently, two non-classically secreted proteins, CLIBASIA_RS00445 and SC2_gp095, were shown to dramatically downregulate the gene expression of RbohB, a gatekeeper of H_2_O_2_-mediated defense signaling in *Nicotiana tabacum* (Jain et al., 2015, 2018), and the former was also identified to suppress oxylipin-mediated defense in citrus plants (Jain et al., 2019). These findings indicate that, apart from the Sec-dependent secretory proteins, CLas also employs non-classically secreted proteins to interfere with the plant defense mechanisms.

*CLIBASIA_04405* was first annotated in CLas strain psy62 (Duan et al., 2009). However, no biological role has been linked to it yet. Here we characterized CLIBASIA_04405 as a Sec-dependent secretory protein of CLas and showed that its mature form (designated m4405) is able to suppress hypersensitive cell death triggered by both the mouse BAX (Lacomme and Santa Cruz, 1999) and the *Phytophthora infestans* INF1 (Kamoun et al., 1998), two well-known hypersensitive response (HR)-based PCD inducers. In addition, we showed that transgenic *N. benthamiana* expressing m4405 displayed improved resistance to *Pseudomonas syringae* DC3000Δ*hopQ1-1* (Wei et al., 2007) as well as defects in growth and development compared with wild-type (WT) *N. benthamiana*. The study revealed an effector of CLas that might contribute to the bacterial pathogenicity.

## Materials and Methods

### *In silico* Analysis

The putative signal peptide (SP) of CLIBASIA_04405, namely, 4405SP, was predicted using SignalP (version 5.0) with default settings for Gram-negative bacteria.

### Alkaline Phosphatase (PhoA) Assay

The coding sequence of 4405SP was amplified with the primers 4405F/4405R (Supplementary Table 1) and cloned into pET-mphoA (Liu et al., 2019) that was double digested with *Nde* I and *Hin*d III. The resulting construct pET-4405SP-mphoA that harbored an in-frame gene fusion between *4405SP* and *mphoA* was transformed into *Escherichia coli* BL21. To detect the alkaline phosphatase (PhoA) activities, the transformants were randomly selected and incubated on the indicator Luria–Bertani agar (90 μg/ml BCIP, 100 mM IPTG, and 75 mM Na_2_HPO_4_) at 37°C, as previously described (Liu et al., 2019). After 24 h of incubation, the transformants with a color change to blue indicate PhoA activity.

### Detection of the Transcripts for CLIBASIA_04405 in CLas-Infected Citrus and Psyllids

Total RNA was extracted from CLas-infected Valencia sweet orange (*Citrus sinensis*) seedlings and CLas-bearing psyllids (Liu et al., 2019). Using the specific primers of *CLIBASIA_04405* (Supplementary Table 1), reverse transcription-quantitative polymerase chain reaction (RT-qPCR) was conducted as previously described (Liu et al., 2019). Three biological replicates were carried out, using the CLas *LasgyrA* gene (GenBank No. CP001677.5) as an internal reference. The relative gene expression was determined using the 2^–Δ^ ^Δ^ ^*Ct*^ method (Livak and Schmittgen, 2001), and statistical analyses were performed using the Student’s *t*-test.

### Subcellular Localization of m4405 in *N. benthamiana*

The coding sequences of CLIBASIA_04405 and m4405 amplified with the specific primers (Supplementary Table 1) were inserted into pCAMBIA1300-35S-GFP digested with *Kpn* I and *Xho* I. The resulting p4405-GFP and pm4405-GFP were individually introduced into *Agrobacterium tumefaciens* EHA105, followed by co-infiltration with agrobacterial cells carrying a plasma membrane marker pm-rk CD3-1007 (Nelson et al., 2007) into leaves of the 4-week-old *N. benthamiana*, as previously described (Van der Hoorn et al., 2000). At 60 h post inoculation (hpi), the infiltrated patches were visualized for GFP fluorescence under a LSM700 confocal microscope (Zeiss, Germany).

### *Agrobacterium*-Mediated PVX Infection Assay

The coding sequence of m4405 amplified with the primers m4405-F/m4405-R (Supplementary Table 1) was cloned into a *Potato virus X* (PVX)-based expression vector pGR107 (Jones et al., 1999) to generate pPVX-m4405. Additionally, nine alanine-substituted mutants of *m4405* (Supplementary Figure 1) were produced by PCR using the primers listed in Supplementary Table 1 and individually inserted into pGR107, resulting in pPVX-A9-10, pPVX-A24-25, pPVX-A39-40, pPVX-A45-47, pPVX-A52-54, pPVX-A59-60, pPVX-A64-65, and pPVX-A75-78, pPVX-A93-94.

The PCD suppression assay was carried out as described previously (Wang et al., 2011). Briefly, the *A. tumefaciens* GV3101 that harbored pPVX-m4405, pPVX-A9-10, pPVX-A24-25, pPVX-A39-40, pPVX-A45-47, pPVX-A52-54, pPVX-A59-60, pPVX-A64-65, pPVX-A75-78, or pPVX-A93-94 were first infiltrated into the expanded leaves of the 6-week old *N. benthamiana* plants. The *A. tumefaciens* cells that harbored pPVX-GFP were included as a negative control. At 24 hpi, the infiltrated patches were further inoculated with the *Agrobacterium* cells that harbored pGR-BAX or pGR-INF1. At 2 days post second inoculation, one third of the infiltrated leaves were detached to perform histochemical staining with 3,3′-diaminobenzidine (DAB) as described (Vanacker et al., 2000). To record the cell death development, the rest leaves were continuously observed up to 5 days post second inoculation. The experiment was repeated three times, and each replicate included six to eight plants.

### Generation of Transgenic *N. benthamiana*

The coding sequence of m4405 was inserted into a binary plasmid vector pBI121 (Chen et al., 2003), resulting in pBI-m4405 that was subsequently introduced into *A. tumefaciens* EHA105. The transgenic *N. benthamiana* lines expressing m4405 were generated using a leaf disk method (Gallois and Marinho, 1995). T1 generation seeds were screened on media containing 50 μg/ml of kanamycin (Kan), and the Kan-resistant seedlings were further confirmed by RT-PCR with the primers listed in Supplementary Table 1.

### *Pseudomonas syringae* Infection Assays

The infection assay was performed as previously described (Wei et al., 2018). DC3000Δ*hopQ1-1* (Wei et al., 2007), a knockout mutant of *P. syringae* pv. *tomato* strain DC3000 was cultured in King’s B (KB) broth with rifampicin (50 μg/ml) at 28°C overnight, and collected by centrifugation (2,500 × *g*, 5 min), followed by washing and resuspension in 10 mM MgCl_2_ to a final concentration of 3 × 10^4^ CFU/ml. The leaves of 4-week-old *N. benthamiana* and transgenic m4405 lines were syringe infiltrated with the bacterial suspensions. At 6 days post inoculation (dpi), the infiltrated leaf tissues were collected and ground in distilled water. Serial 10-fold dilutions were spread on KB plates with rifampicin (50 μg/ml) and incubated at 28°C for 2 days. Bacterial counts were expressed as CFU/cm^2^ leaf disc and represented the mean ± SD (standard deviation) of three biological replicates. Statistical analysis was determined using Student’s *t*−test.

### Transcriptome Sequencing and Analysis of Differentially Expressed Genes

Total RNA was extracted from leaves of the 4-week-old *N. benthamiana* and transgenic m4405 lines by using an RNeasy Mini kit (Qiagen, United States), followed by cDNA library preparation and in-depth sequencing using an Illumina HiSeq2000. The resulting clean reads were assembled into transcripts using the Trinity method (Grabherr et al., 2011). After removing redundancy, the unigenes were generated and subjected to a blast search against the public protein databases of NR (NCBI non-redundant protein sequences), Swiss-Prot, NT (NCBI nucleotide sequences), and Protein family (Pfam) by using NCBI blastX with a cutoff *E*-value of ≤1e-5. The clean reads from *N. benthamiana* and the transgenic m4405 lines were individually mapped to the assembled transcriptome using RSEM with no more than two nucleotide mismatches (Li and Dewey, 2011), and the resulting read count for each matched unigene was further normalized using the reads per kilo bases per million read (RPKM) method (Mortazavi et al., 2008). The differentially expressed genes (DEGs) were determined using DEGseq (Wang et al., 2010) with threshold as “*q*-value < 0.005 and | log2 (foldchange)| > 1.” Heatmap was created using the function heatmap.2 of gplots R package (Warnes et al., 2015). The nucleotide sequences of raw reads and the assembled unigene were submitted to NCBI Sequence Read Archive (SRA) database under the accession number PRJNA663964.

### Detection of the Transcripts for sHSPs in the Transgenic m4405 *N. benthamiana*

Total RNA from leaves of the 4-week-old *N. benthamiana* and transgenic m4405 lines was subjected to RT-qPCR analysis as previously described (Zhang et al., 2019). The gene-specific primers used are listed in Supplementary Table 1. The experiment was performed with three biological replicates, using the *N. benthamiana actin* (GenBank No. JQ256516.1) as a reference gene. The relative gene expression was determined using the 2^–Δ^ ^Δ^ ^*Ct*^ method (Livak and Schmittgen, 2001), and statistical analysis was conducted using Student’s *t*-test.

## Results

### CLIBASIA_04405 Was a Sec-Dependent Secretory Protein That Exhibited Higher Transcriptional Level in Citrus Than in Psyllids

**FIGURE 1:**
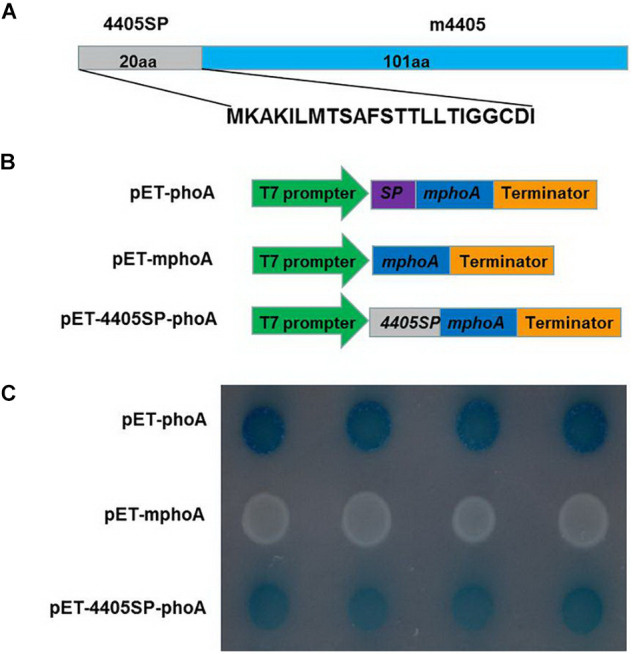
Experimental validation of the signal peptide of CLIBASIA_04405. **(A)** The primary structure of CLIBASIA_04405. The putative signal peptide was termed 4405SP, and the mature form was denoted as m4405. **(B)** Schematic diagrams of the *phoA* gene expression cassettes in pET-phoA, pET-mphoA, and pET4405sp-mphoA. pET-phoA bearing the full length *phoA* gene was used as a positive control, whereas pET-mphoA that harbors *mphoA* lacking the native SP-coding sequence was used as a negative control. **(C)** 4405SP directed the extracytoplasmic secretion of mPhoA. The bacterial cells were incubated at 37°C on the indicator LB media that contain Na_2_HPO_4_ (75 mM), IPTG (100 mM), and BCIP (90 μg/ml). The cells harboring pET-phoA (the positive control) or pET-4405sp-mphoA quickly turned dark blue, whereas the cells bearing pET-mphoA (the negative control) remain white at 24 h of incubation. The photo was taken at 6 h of incubation.

The CLIBASIA_04405 of CLas is composed of 121 amino acids (aa) (Duan et al., 2009). A BLAST search of the GenBank database revealed that this protein was 100% conserved among the CLas strains with the full genome available (Supplementary Figure 2), but was absent from both CLaf and CLam. An *in silico* analysis with SignalP indicated that CLIBASIA_04405 had 4405SP, a 20-aa SP at its N-terminus (Figure 1A). To functionally validate 4405SP, the *E. coli*-based phoA fusion assay was performed based on the chromogenic reaction of the secreted PhoA protein on LB medium containing BCIP substrate (Liu et al., 2019). After 24 h of incubation, the BL21 cells that expressed mphoA lacking its native SP, which served as a negative control, remained white, whereas the bacteria that expressed 4405SP-mPhoA turned dark blue (Figures 1B,C) after 6 h of incubation, indicating that 4405SP successfully directed the extracellular secretion of the mPhoA moiety. The *in silico* prediction combined with the experimental validation supported the concept that CLIBASIA_04405 had an SP that could direct the protein secretion through the Sec-dependent pathway.

**FIGURE 2:**
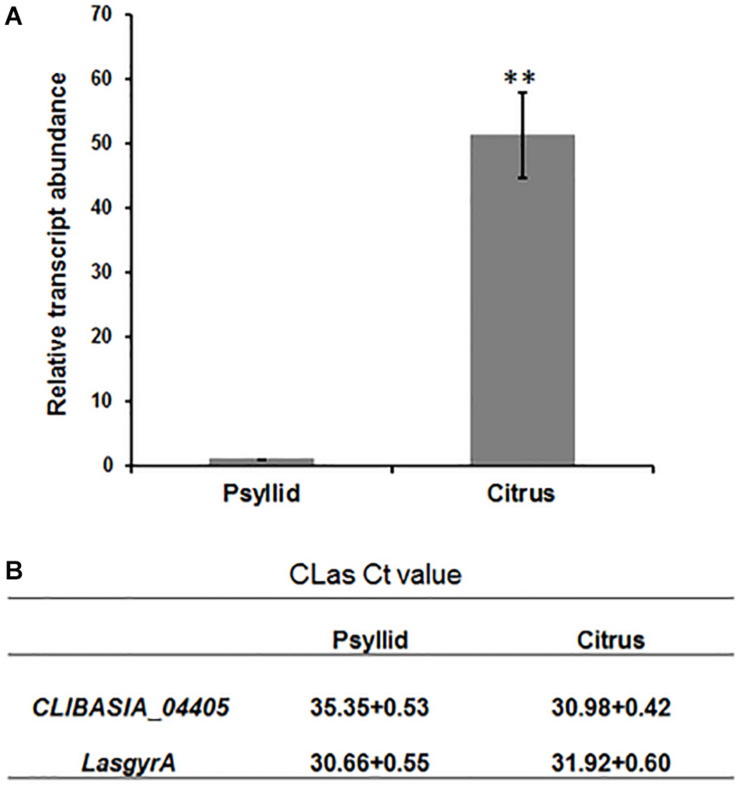
RT-qPCR analysis of gene expression of CLIBASIA_04405 in psyllid and citrus. **(A)** Relative transcript abundance of *CLIBASIA_04405* in psyllid and citrus. **(B)** The Ct values of *CLIBASIA_04405* and the internal reference gene *LasgyrA*. Bars represent standard error of the means; asterisks indicate a significant difference (Student’s *t*-test, **p < 0.01).

We next detected the transcripts for CLIBASIA_04405 in host plants and insect vectors. RT-qPCR analysis was performed on total RNA extracted from the CLas-infected sweet orange and ACPs, and the result showed that the gene expression level of CLIBASIA_04405 in citrus was significantly higher (∼50-fold) than that in psyllids (Figures 2A,B).

### m4405 Was Localized in Plasma Membrane of *N. benthamiana* Cells

**FIGURE 3:**
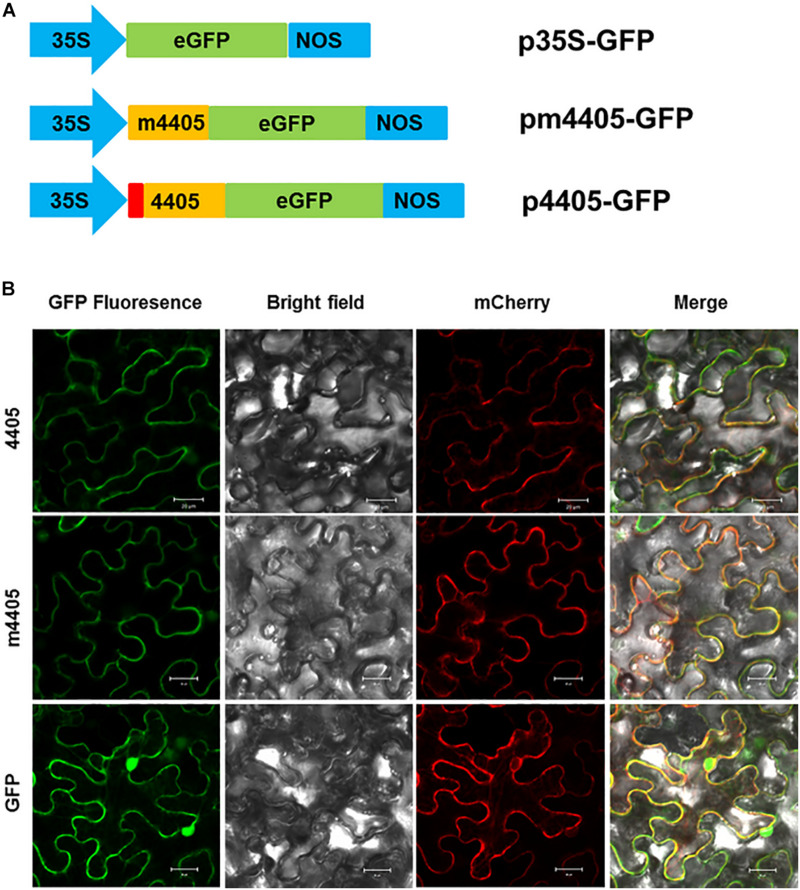
Subcellular localization of m4405 in *Nicotiana benthamiana*. **(A)** Schematic representation of the expression cassettes for green fluorescent protein (GFP), m4405-GFP and 4405-GFP. CaMV 35S promoter is indicated as 35S, and nopaline synthase terminator is denoted as NOS. **(B)** Fluorescence of m4405-GFP and 4405-GFP in leaf epidermal cells of *N. benthamiana*. pm-rk *CD3*-1007 was used as a marker for plasma membrane. Fluorescent signals were observed under confocal microscopy by using the following settings: GFP, 500–550 nm; mCherry, 600–650 nm. Scale bars represent 20 μm.

Since CLIBASIA_04405 was a Sec-dependent secretory protein, we investigated the subcellular localization of its mature protein m4405 in plants. The m4405 coding sequence was fused with the GFP gene (Figure 3A) and expressed in *N. benthamiana* via *Agrobacterium*-mediated transient expression. The N-terminal GFP-tagged m4405 was detected under confocal fluorescence microscopy. As shown in Figure 3B, m4405-GFP was predominately colocalized with the plasma membranal marker pm-rk *CD3*-1007 (Nelson et al., 2007), indicating the plasma membrane localization of m4405 in plants. Using the same strategy, we observed that CLIBASIA_04405 shared a similar subcellular localization in *N. benthamiana* cells with m4405 (Figure 3B). This suggested that the SP of CLIBASIA_04405 had little or no effect on plasma membrane localization of the protein in plants.

### m4405 Suppressed BAX- and INF1-Triggered Hypersensitive Cell Death in *N. benthamiana*

**FIGURE 4:**
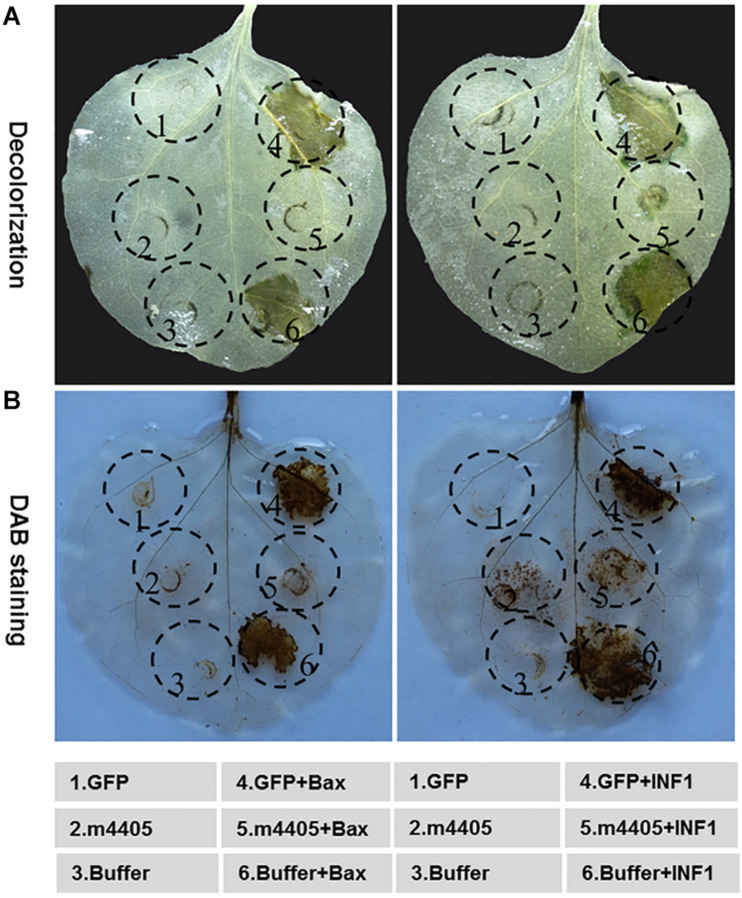
Cell death suppression of m4405. **(A)** The m4405 suppressed the hypersensitive cell death triggered by BAX and INF1 in *Nicotiana benthamiana*. The inoculation buffer or *Agrobacterium* cells containing *m4405* or *GFP* were infiltrated into the leaves of *N. benthamiana*, either alone or followed 24 h later with *Agrobacterium* cells carrying *BAX* or *INF1*. The leaves were detached at 5 days post the *BAX*- or *INF1* infiltration, followed by decolorization with ethanol. **(B)** The m4405 inhibited overaccumulation of H_2_O_2_ induced by BAX and INF1 in *N. benthamiana*. Agroinfiltration was conducted as mentioned above, and the leaves were collected at 2 days post *BAX*- or *INF1* infiltration and stained with 3,3′-diaminobenzidine (DAB).

On the basis of a plant virus expression vector pGR107 (Jones et al., 1999), the mouse BAX and the *P. infestans* INF1 have been widely utilized to identify the PCD suppressor of phytopathogens (Bos et al., 2006; Li et al., 2015; Xiang et al., 2016; Fang et al., 2019). We employed this strategy to evaluate the ability of m4405 in PCD suppression. As shown in Figure 4A, m4405 completely suppressed both BAX- and INF1-triggered HR-based cell death in *N. benthamiana*, whereas cell death suppression had not been observed for GFP and the infiltration buffer.

To further understand the role of m4405 that leads to cell death suppression, the accumulation of H_2_O_2_, a reactive oxygen species (ROS) critical in plant cell death (Petrov and Van Breusegem, 2012), was analyzed in the *N. benthamiana* leaves co-expressing m4405 plus either BAX or INF1. Using a DAB staining method (Vanacker et al., 2000), we detected that m4405 completely inhibited both BAX- or INF1-stimulated H_2_O_2_ burst (Figure 4B). By contrast, neither GFP nor the infiltration buffer interfered with H_2_O_2_ accumulation.

To identify the key domain(s) of the m4405 involved in PCD suppression, nine clusters of charged amino acids (ED^9–10^, KE^24–25^, ER^39–40^, EKR^45–47^, EER^52–54^, RK^59–60^, DE^64–65^, DEDK^75–78^, and KK^93–94^) of m4405 were individually selected for alanine-substitution mutagenesis (Supplementary Figure 1). The resulting mutants were expressed through pGR107 to test their abilities of PCD suppression. As results, there were two mutants (A45-47 and A64-65), whose abilities to suppress PCD induced by INF1 and BAX were both greatly reduced or even lost completely (Table 1), indicating that EKR^45–47^ and DE^64–65^ are necessary for m4405 ability to suppress cell death.

**TABLE 1:** Identification of the key amino acids of m4405 involved in PCD suppression.

Mutant^*a*^	PCD Inducer^*b*^	
	BAX	INF1
m4405	0/135	0/99
GFP	135/135	99/99
A9-10	1/45	9/33
A24-25	1/45	21/33
A39-40	1/45	22/33
A45-47	45/45	32/33
A52-54	0/45	30/33
A59-60	0/45	12/33
A64-65	22/45	31/33
A75-78	6/45	16/33
A93-94	10/45	28/33

*^a^The m4405 and the alanine-substituted m4405 mutants were transiently expressed in Nicotiana benthamiana via Agroinfiltration. ^b^The mouse BAX and the Phytophthora infestans INF1 were used as PCD inducers. The development of cell death was recorded at 5 days post infiltration of BAX or INF1. The PCD suppression of m4405 and its related mutants are shown as “lesion number/infiltration number.”*

### The Transgenic *N. benthamiana* Expressing m4405 Exhibited Improved Resistance to *P. syringae*

**FIGURE 5:**
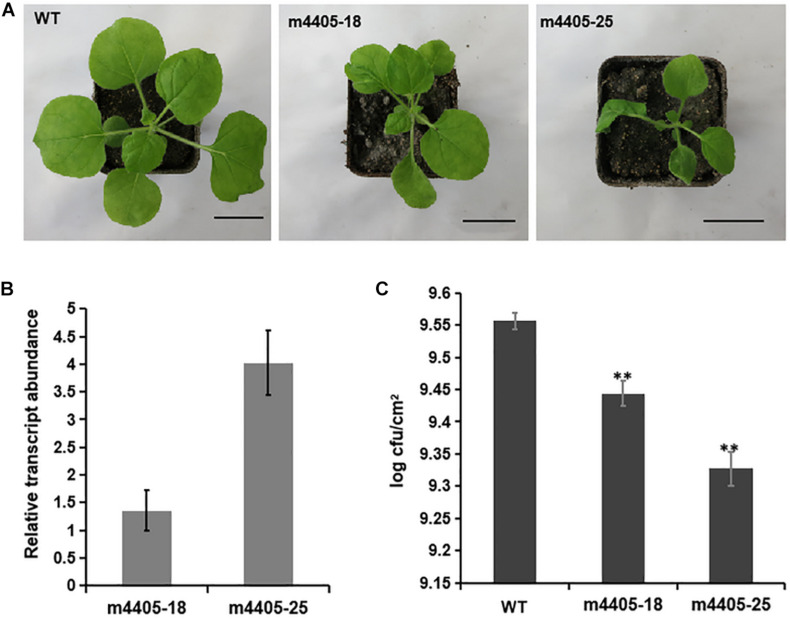
The characteristics of the transgenic *Nicotiana benthamiana* expressing m4405. **(A)** Phenotypes of the *transgenic N. benthamiana*. The m4405-18 and m4405-25 represent the transgenic lines with typical phenotypes of dwarfism and leaf deformation. Scale bars represent 3 cm. **(B)** Transcriptional levels of *m4405* in the transgenic *N. benthamiana*. RT-qPCR was conducted on total RNA prepared from the leaves of m4405-18 and m4405-25. Bars represent standard error of the means. **(C)** Bacterial growth in leaves of the transgenic m4405 lines and *N. benthamiana*. *Pseudomonas syringae* DC3000Δ*hopQ1-1* was infiltrated at 10^4^ CFU ml^– 1^, and populations were measured from leaf discs at 6 days after inoculation. Bars represent standard error of the mean, and asterisks indicate the significant differences (Student’s *t*-test, **p < 0.01).

To gain further insight into the impact of m4405 on plant immunity, we generated transgenic *N. benthamiana* that stably expressed m4405 under the control of *Cauliflower mosaic virus* (CaMV) 35S promoter. A total of six independent transgenic lines were produced, and each of them exhibited phenotypes of dwarfism and leaf malformation. The lines m4405-18 and m4405-25 (Figure 5A) were then randomly selected for the following experiments. In the first experiment, RT-qPCR analysis was performed on total RNA of transgenic *N. benthamiana* and disclosed that the expression level of m4405 positively correlated with the phenotypic changes of the transgenic lines (Figures 5A,B). Second, using *N. benthamiana* as a control, the transgenic plants were challenged with DC3000Δ*hopQ1-1*, a *P. syringae* DC3000 mutant that lacks the type III effector HopQ1-1 and thus is able to grow in *N. benthamiana* instead of inducing an HR reaction (Wei et al., 2007, 2013). Measurement of growth of DC3000Δ*hopQ1-1* in the inoculated leaf tissues at 6 dpi showed that the transgenic m4405 plants supported significantly less bacterial proliferation than WT (Figure 5C), indicating that the transgenic plants exhibited improved resistance to DC3000Δ*hopQ1-1*.

**FIGURE 6:**
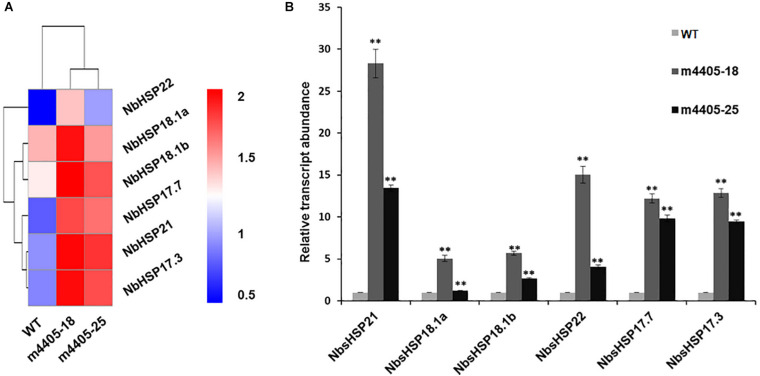
The *sHSP* genes were significantly upregulated in the transgenic *N. benthamiana* expressing m4405. **(A)** Heatmap representation of the normalized gene expression values of six small heat shock proteins (sHSPs) in *N. benthamiana* and the transgenic m4405 lines. **(B)** Validation of the expression of six *sHSP* genes by RT-qPCR. The transcript abundance of the indicated gene was normalized against the expression of *Nbactin*. Bars represent the standard error, and asterisks indicate the significant differences (Student’s *t*-test, **p < 0.01).

To address the underlying mechanism of resistance to DC3000Δ*hopQ1-1* in the transgenic m4405 lines, we compared the transcriptome profiles of m4405-18 and m4405-25 with that of WT and identified an array of DEGs (Supplementary Table 2). Mining the DEGs revealed that a subset of genes encoding small heat shock proteins (sHSPs), including sHSP17.7, sHSP17.3, sHSP18.1a, sHSP18.1b, sHSP21, and sHSP22, were significantly upregulated in the transgenic lines compared with WT (Figure 6A). RT-qPCR analysis confirmed that the mRNAs for the six sHSPs were significantly accumulated in the transgenic lines (Figure 6B). The sHSPs are known to be a group of proteins with a molecular mass of 15 to 42 kDa that act as molecular chaperones to protect the plants against various biotic and abiotic stress conditions (Haslbeck and Vierling, 2015). Taking these results together, we inferred that the upregulated sHSPs in the transgenic m4405 *N. benthamiana* could contribute to the increased resistance to DC3000Δ*hopQ1-1*.

## Discussion

Although the molecular basis of the CLas pathogenicity remains largely unknown, much progress has been made toward elucidating its mechanisms of pathogenesis, particularly through the identification and characterization of CLas-encoded effectors, such as the Sec-dependent secretory proteins (Pitino et al., 2016, 2018; Clark et al., 2018; Liu et al., 2019; Shi et al., 2019; Ying et al., 2019; Zhang et al., 2019; Li et al., 2020). In a previous study, a genome-wide *in silico* analysis on CLas psy62 strain predicted a total of 166 proteins containing SPs that may target them out of the cytoplasm via the Sec-dependent pathway (Prasad et al., 2016). However, 51 of these proteins, including CLIBASIA_04405, were determined to be false-positive predictions (Prasad et al., 2016) when experimentally validated using an early developed phoA fusion system (Mdluli et al., 1995). In this study, we revisited the putative SP of CLIBASIA_04405 with an updated phoA system (Liu et al., 2019) and provided evidence that CLIBASIA_04405 was instead a Sec-dependent secretory protein of CLas. Furthermore, we found that the expression level of *CLIBASIA_04405* was significantly higher in a citrus host than in insect vectors. It is worthy to emphasize that a similar expression pattern was also observed in several CLas genes, such as CLIBASIA_00255, SC2_gp095, CLIBASIA_RS00445, CLIBASIA_00460, and CLIBASIA_03875, which have been suggested to function in the context of the CLas infection on the host plants (Jain et al., 2015, 2018; Li et al., 2017; Liu et al., 2019; Zhang et al., 2019). In addition, we show that m4405, the mature form of CLIBASIA_4405, completely suppresses PCD triggered by both BAX and INF1 in *N. benthamiana*. The data collectively indicates that CLIBASIA_4405 served as a virulence factor and was implicated in CLas pathogenesis in plant.

Programmed cell death plays roles in multiple cellular processes of plants, including immunity (Huysmans et al., 2017; Locato and De Gara, 2018). Upon pathogen infection, the plants may prime a form of PCD termed HR, a strong immune response that is accompanied by localized cell death to prevent the spread of the pathogen (Coll et al., 2011; Stael et al., 2015). In contrast, the pathogens, including bacteria, fungi, oomycetes, and nematodes, often secrete an arsenal of effectors that contribute to cell death suppression to enable their successful infection (da Cunha et al., 2007; Dou and Zhou, 2012; Goverse and Smant, 2014; Lo Presti et al., 2015). In CLas, the mature forms of two Sec-dependent secretory proteins CLIBASIA_03875 and CLIBASIA_04025, termed m3875 and SDE15, have been determined as PCD suppressors (Zhang et al., 2019; Pang et al., 2020). Herein, we demonstrated that m4405, the mature form of the Sec-dependent secretory protein CLIBASIA_04405, also inhibited hypersensitive cell death. Together, the findings highlight that, despite lacking the conserved secretion machineries such as T3SS (Duan et al., 2009), CLas employs the Sec-dependent secretory proteins to overcome HR of the citrus host.

To inhibit the development of hypersensitive cell death, pathogen-encoded PCD suppressors may interact with the host immune receptors (Bos et al., 2010; Wilton et al., 2010; Chen et al., 2012), or interfere with ROS accumulation that accompanies cell death (Dong et al., 2011; Hemetsberger et al., 2012; Zhang et al., 2015a). It has been shown that the CLas PCD suppressor m3875 regulates the gene expression of cell death regulators, cyclic nucleotide-gated channels (CNGC), Bax-inhibitor 1 (BI-1), and WRKY9 (Zhang et al., 2019), while another CLas PCD suppressor SDE15 directly interacts with a citrus protein ACCELERATED CELL DEATH 2 (ACD2), a negative regulator of PCD (Pang et al., 2020). In this study, how m4405 suppressed hypersensitive cell death remains elusive. However, alanine-substitution mutagenesis of m4405 showed that two amino acid clusters (EKR^45–47^ and DE^64–65^) were crucial for suppressing both BAX- and INF1-triggered cell death. Further study on the two loss-of-function mutants (A45-47 and A64-65) as well as identification of the potential target protein(s) in plants could be helpful to elucidate the mechanism by which m4405 suppressed PCD.

A number of pathogen effectors have been shown to mediate disease resistance when expressed transgenically (Kawamura et al., 2009; Rajput et al., 2015; Zhang et al., 2015b; Wang et al., 2019). Likewise, the study here displayed that the transgenic *N. benthamiana* expressing m4405 exhibited improved resistance to the bacterial pathogen DC3000Δ*hopQ1-1*. Transcriptome profiling and RT-qPCR revealed that the gene expression of six sHSPs (sHSP17.7, sHSP17.3, sHSP18.1a, sHSP18.1b, sHSP21, and sHSP22) were significantly upregulated in the transgenic lines. The sHSPs are evolutionarily conserved proteins in almost all organisms, ranging from bacteria and fungi to plants and animals (Waters and Vierling, 1999; Haslbeck and Vierling, 2015). The plant sHSPs are usually below detection limits in vegetative tissues under normal growth conditions but instead are induced by environmental stresses and developmental stimuli (Jacob et al., 2017; Carra et al., 2019). So far, there is emerging evidence that sHSPs serve as defense regulators and function in diverse defense mechanisms (Ul Haq et al., 2019). For example, the *N. tabacum* sHSP17 is required for basic immune responses other than HR-based resistance in plants (Maimbo et al., 2007). In contrast, sHSP20s plays a positive role in the hypersensitive defense response, and silencing of *HSP20s* in *N. benthamiana* can compromise the HR induced by the tomato resistance (*R*) genes *I-2* and *Mi-1* (Van Ooijen et al., 2010). In addition, a subset of the *Arabidopsis* sHSPs, including sHSP22, has been identified to be downregulated upon *Pst* DC3000 infection (Bricchi et al., 2012), implying their functions in response to the *P. syringae* infection. Remarkably, sHSP22 was one of the six sHSPs that were transcriptionally activated in the transgenic m4405 *N. benthamiana*. The potential of sHSP22, as well as those of the other five sHSPs, in *P. syringae* resistance merits further investigation.

In conclusion, the study identified a Sec-dependent secretory protein of CLas and showed that its mature form m4405 could suppress hypersensitive cell death in *N. benthamiana*. Transgenic overexpression of m4405 in *N. benthamiana* interfered with the plant growth and development and, in particular, upregulated the transcription of a group of *sHSP* genes. The data collectively indicated the potential role of m4405 in CLas infection and might shed new light on the pathogenicity of CLas.

## Data Availability Statement

The datasets presented in this study can be found in online repositories. The names of the repository/repositories and accession number(s) can be found in the article/Supplementary Material.

## Author Contributions

WL, XW, and CZ designed the experiments. PD, HY, CZ, and ZZ performed the experiments. WL and CZ analyzed the data. WL, CZ, and XW wrote the manuscript. All authors contributed to the article and approved the submitted version.

## Conflict of Interest

The authors declare that the research was conducted in the absence of any commercial or financial relationships that could be construed as a potential conflict of interest. The reviewer PY declared a shared affiliation with several of the authors, CZ, PD, XW, and WL, to the handling editor at the time of review.
