# Supplementary material for: A Sec-Dependent Secretory Protein of the Huanglongbing-Associated Pathogen Suppresses Hypersensitive Cell Death in Nicotiana benthamiana
Source: Front Microbiol. 2020 Nov 30;11:594669. doi: 10.3389/fmicb.2020.594669 (PMC7734103; doi:10.3389/fmicb.2020.594669)
Supplement: Supplementary file 1 [file Image_1.PDF]

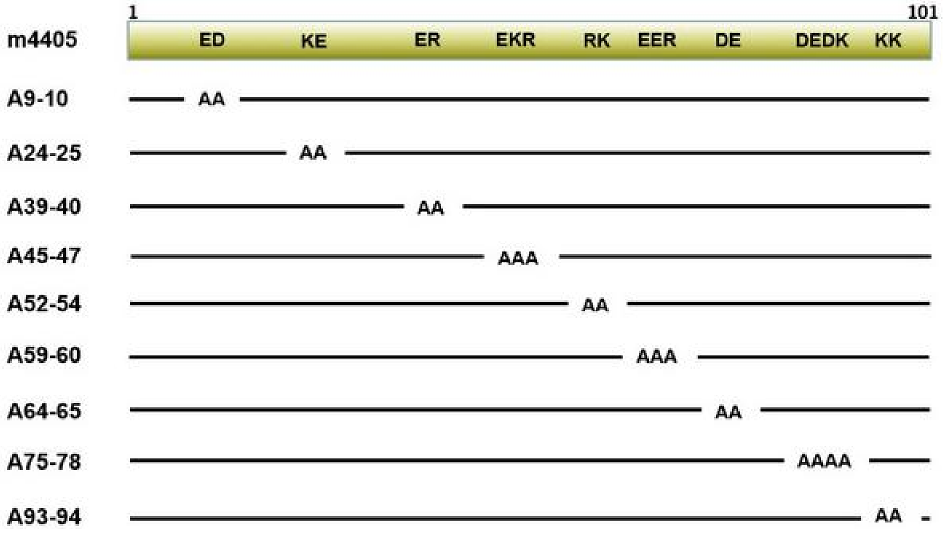

Figure S1. Schematic diagrams of the alanine-substituted m4405 mutants. Nine charged amino acid clusters in m4405 were individually substituted with alanines.
