# Supplementary material for: A Sec-Dependent Secretory Protein of the Huanglongbing-Associated Pathogen Suppresses Hypersensitive Cell Death in Nicotiana benthamiana
Source: Front Microbiol. 2020 Nov 30;11:594669. doi: 10.3389/fmicb.2020.594669 (PMC7734103; doi:10.3389/fmicb.2020.594669)
Supplement: Supplementary file 2 [file Image_2.PDF]

```
psy62      MKAKILMTSA FSTTLLTIGG CDIVIGRTED LLNKLQKNST QMIKEANFKI
lshi-1     MKAKILMTSA FSTTLLTIGG CDIVIGRTED LLNKLQKNST QMIKEANFKI
AHCA1      MKAKILMTSA FSTTLLTIGG CDIVIGRTED LLNKLQKNST QMIKEANFKI
JXGC       MKAKILMTSA FSTTLLTIGG CDIVIGRTED LLNKLQKNST QMIKEANFKI
A4         MKAKILMTSA FSTTLLTIGG CDIVIGRTED LLNKLQKNST QMIKEANFKI
gxpsy      MKAKILMTSA FSTTLLTIGG CDIVIGRTED LLNKLQKNST QMIKEANFKI

psy62      SETHRLAQER VEAAEKRVKE VEERATASRK LSVDELANAF WDLSDEDKNA
lshi-1     SETHRLAQER VEAAEKRVKE VEERATASRK LSVDELANAF WDLSDEDKNA
AHCA1      SETHRLAQER VEAAEKRVKE VEERATASRK LSVDELANAF WDLSDEDKNA
JXGC       SETHRLAQER VEAAEKRVKE VEERATASRK LSVDELANAF WDLSDEDKNA
A4         SETHRLAQER VEAAEKRVKE VEERATASRK LSVDELANAF WDLSDEDKNA
gxpsy      SETHRLAQER VEAAEKRVKE VEERATASRK LSVDELANAF WDLSDEDKNA

psy62      FTGNVKQEVC KVKKITVPPS N
lshi-1     FTGNVKQEVC KVKKITVPPS N
AHCA1      FTGNVKQEVC KVKKITVPPS N
JXGC       FTGNVKQEVC KVKKITVPPS N
A4         FTGNVKQEVC KVKKITVPPS N
gxpsy      FTGNVKQEVC KVKKITVPPS N
```

Figure S2. ClustalW alignment of the protein sequences of CLIBASIA_04405 from six CLas strains for which the complete genome or chromosome was available, including psy62 (CP001677.5), lshi-1 (AP014595.1), AHCA1 (CP029348.1), JXGC (CP019958.1), A4 (CP010804.2) and gxpsy (CP004005.1).
