# Supplementary material for: A Sec-Dependent Secretory Protein of the Huanglongbing-Associated Pathogen Suppresses Hypersensitive Cell Death in Nicotiana benthamiana
Source: Front Microbiol. 2020 Nov 30;11:594669. doi: 10.3389/fmicb.2020.594669 (PMC7734103; doi:10.3389/fmicb.2020.594669)
Supplement: Supplementary file 3 [file Table_1.DOC]

Table S1. The primers used in this study^1^

| **Application** | **Primer name** | **Primer sequence (5＇-3＇)** |
| --- | --- | --- |
| **Constructs preparation**  pET-4405SP-mphoA  pm4405-GFP  p4405-GFP  pPVX-m4405  pPVX-eGFP  pPVX-A9-10  pPVX-A24-25  pPVX-A39-40  pPVX-A45-47    pPVX-A52-54  pPVX-A59-60  pPVX-A64-65  pPVX-A75-78  pPVX-A93-94  **RT-qPCR assay**  *Nbactin*  *NbsHSP21*  *NbsHSP18.1a*  *NbsHSP18.1b*  *NbsHSP22*  *NbsHSP17.7*  *NbsHSP17.3* | 4405SP-F  4405SP-R  m4405gfp-F  m4405gfp-R  4405gfp-F  m4405gfp-R  m4405-F  m4405-R  eGFP-F  eGFP-R  4405m1-CLAI-F  m4405-R  m4405m2-F  m4405m2-R  m4405m3-F  m4405m3-R  m4405m4-F  m4405m4-R  m4405m5-F  m4405m5-R  m4405m6-F  m4405m6-R  m4405m7-F  m4405m7-R  m4405m8-F  m4405m8-R  m4405-F  m4405m9-R  OligodT  Nbactin-F  Nbactin-R  NbsHSP21-F  NbSHSP21-R  NbsHSP18.1a-F  NbSHSP18.1a-R  NbsHSP18.1b-F  NbSHSp18.1b-R  NbsHSP22-F  NbSHSP22-R  NbsHSP17.7-F  NbSHSP17.7-R  NbsHSP17.3-F  NbSHSP17.3-R | AAGGAGATATA**CATATG**AAAGCTAAAATATTGATGAC  AGGCATTTCTGG**AAGCTT**ATGACAATGTCACAACCAC  GACGAGCTCG**GGTACC**ATGTGTGACATTGTCATTGGTAG  TGGTGTCGAC**TCTAGA**ATTTGATGGGGGCACAGTGA  GACGAGCTCG**GGTACC**ATGAAAGCTAAAATATTGATGAC  ----------------------  GAGCACCAGCTAGC**ATCGAT**ATGTGTGACATTGTCATTGG  CAAGCTTATCGGCGG**TCGAC**CTAATTTGATGGGGGCACAG  GAGCACCAGCTAGC**ATCGAT**ATGGTGAGCAAGGGCGAGGA  CAAGCTTATCGGCG**GTCGAC**TCAAAGATCTACCATGTACA  GCACCAGCTAGC**ATCGAT**atgTGTGACATTGTCATTGGTAGAACAGcGGcCCTTTTG  -----------------------  ACGCAAATGATTgcGGcAGCTAATTTTAA  TTAAAATTAGCTgCCgcAATCATTTGCGT  GATTAGCACAGGcAgcTGTTGAGGCGGC  GCCGCCTCAACAgcTgCCTGTGCTAATC  TTGAGGCGGCGGcAgctgcAGTGAAAGAAGT  ACTTCTTTCACTgcagcTgCCGCCGCCTCAA  TGAAAGAAGTCGcAGcAgcCGCTACTGCATC  GATGCAGTAGCGgcTgCTgCGACTTCTTTCA  GCTACTGCATCGgcAgcACTATCAGTAG  CTACTGATAGTgcTgcCGATGCAGTAGC  ACTATCAGTAGcTGcATTGGCGAACGCT  AGCGTTCGCCAATgCAgCTACTGATAGT  GGGATTTGAGTGcTGcAGcCgcAAACGCTTTTAC  GTAAAAGCGTTTgcGgCTgCAgCACTCAAATCCC  ----------------------------  AGCTTATCGGCG**GTCGAC**CTAATTTGATGGGGGCACAGTGATcgcTgcAACCTTG  TTTTTTTTTTTTTTTTTT  CCTGAGGTCCTTTTCCAACCA  GGATTCCGGCAGCTTCCATT  TTCGCTCTGCCTCTGTTGCT  GTTGACTCACGCTCCTTGGT  TGTTCCATCTTCTGCTCGTGA  GCCACTGCTCCTTTCCATAC  AACCTCTGCCTTCGCTAACG  TTTGCCGCTGCTCCTTTCT  CAGAGTGTTGAGAATTAGCGG  GCAGCATTCCCTTCCTCAGA  AGACGATTCAGGCTCCCAGA  ACATCAGGCTTCTTCACCTCT  ACACTCTCCAGCACCTTATGG  TCTCTCCACTAATCAGCAGCA |

1. The restriction recognition sequences of *Nde* I (CATATG), *Hin*d III (AAGCTT), *Kpn* I (GGTACC), *Xba* I (TCTAGA), *Cla* I (ATCGAT) and *Sal* I (GTCGAC) within the primers are in bold and underlined. Genes included in qPCR assay are *Nbactin* (GenBank No. JQ256516.1) and NbsHSP21 (Niben101Scf04091g00013.1), NbsHSP18.1a (Niben101Scf09262g01017.1), NbSHSp18.1b (Niben101Scf01475g00019.1), NbSHSP22 (Niben101Scf00705g04009.1), NbSHSP17.7 (Niben101Scf09973g00011.1), NbSHSP17.3 (Niben101Scf03114g03011.1) from *N. benthamiana* draft genome sequence V1.0.1 (https://solgenomics.net/organism/Nicotiana_benthamiana/genome).
